# Influence of Mouthwash Rinsing on the Mechanical Properties of Polymeric Ligature Ties Used for Dental Applications

**DOI:** 10.3390/polym13142236

**Published:** 2021-07-08

**Authors:** Aphinan Phukaoluan, Anak Khantachawana, Surachai Dechkunakorn, Niwat Anuwongnukroh, Phacharaphon Tunthawiroon, Kasama Srirussamee

**Affiliations:** 1 Department of Mechanical Technology Education, Faculty of Industrial Education and Technology, King Mongkut’s University of Technology Thonburi (KMUTT), Bangkok 10140, Thailand; aphinan.phu@kmutt.ac.th; 2 Department of Mechanical Engineering, Faculty of Engineering, King Mongkut’s University of Technology Thonburi (KMUTT), Bangkok 10140, Thailand; anak.kha@kmutt.ac.th; 3 Biological Engineering Program, Faculty of Engineering, King Mongkut’s University of Technology Thonburi (KMUTT), Bangkok 10140, Thailand; 4 Department of Orthodontics, Faculty of Dentistry, Mahidol University, Bangkok 10400, Thailand; dtsdh@mahidol.ac.th (S.D.); dtnan@mahidol.ac.th (N.A.); 5 Department of Industrial Engineering, School of Engineering, King Mongkut’s Institute of Technology Ladkrabang (KMITL), Bangkok 10520, Thailand; phacharaphon.tu@kmitl.ac.th; 6 Department of Biomedical Engineering, School of Engineering, King Mongkut’s Institute of Technology Ladkrabang (KMITL), Bangkok 10520, Thailand

**Keywords:** polymeric orthodontic ligature ties, mouthwashes, mechanical properties

## Abstract

Mouthwashes are used during dental treatments to mitigate the complications caused by poor oral hygiene. However, these solutions also affect the properties of dental appliances, including those used in orthodontics. This point has been investigated in this study focusing on the changes in mechanical properties of polymeric orthodontic ligature ties. Commercial ties from four brands were characterized in terms of their maximum forces and displacement, delivery forces, molecular structures, and microscopic morphology. These properties were compared against the ties, which were rinsed with commercial mouthwashes from three manufacturers. The results showed that mouthwash rinsing significantly reduced the maximum bearable forces of ligature ties by up to 73.1%, whereas the reduction in their maximum displacement was up to 74.5% across all tested brands. Significant changes in microscopic morphology of ligature ties were observed after mouthwash rinsing, but not their molecular structure. Furthermore, mouthwash rinsing also reduced the delivery forces from ligature ties by between 20.9 and 32.9% at their first deformation cycle. It can be concluded from this study that mouthwashes have significant impact on the mechanical properties of polymeric orthodontic ligature ties and could also potentially affect the overall efficacy of orthodontic and other dental treatments.

## 1. Introduction

Orthodontic treatments are clinical procedures carried out by the dentists to relocate or correct the misaligned teeth or jaws, which use orthodontic appliances to induce tissue movement, such as arch wires, brackets, screws, ligature ties, elastic bands, and chains [1,2,3,4,5,6,7,8,9,10]. However, the installation of orthodontic appliances could also lead to the accumulation of microorganisms within the oral cavity, including *Streptococcus mutans*, *Lactobacilli*, and *Candida* [11,12,13]. These microorganisms produce acidic compounds which can cause complications during orthodontic treatments, such as tooth decay and demineralization [11,12,13]. As a result, antimicrobial mouthwashes are often used to mitigate this issue [14,15].

It appears that the use of mouthwashes does not only affect the presence of microorganisms, but also the properties of orthodontic appliances. It was reported that the use of mouthwashes could increase the friction of stainless steel and titanium arch wires when sliding through the brackets, which negatively affected the efficacy of orthodontic treatments, and sodium fluoride was reportedly responsible for these changes [7,16,17,18]. On the contrary, this was not the case for tensile strength of stainless steel arch wires, as no significant change was found after being exposed to mouthwash [19]. Furthermore, the delivery force of orthodontic elastomeric chains and tensile strength of O-rings used for overdenture attachment were also negatively affected by mouthwashes [6,20].

Nonetheless, it is still unclear whether or not the changes in mechanical properties of orthodontic ligature ties upon the exposure to mouthwash are significant in practice. These ligature ties are used to hold the position of arch wire in place located at the designated area of the bracket in order to optimize the induction of tooth movement, which has contributed up to 3% of the total displacement in some cases [1,2,3,4]. Ligature ties can be made of metals and elastomers, though the elastomeric type was reported to be easier and quicker to handle [1,2,3]. Changes in the delivery forces of ligature ties would alter the tooth movement and orthodontic treatment efficacy, and these changes were dependent on material, ligation method, and also the oral condition [1,2,3]. Previous studies have reported that the delivery forces of polymeric orthodontic ligature ties reduced dramatically following the first 24 h of their usage due to their viscoelastic properties [1,21,22]. However, it has not been thoroughly investigated whether mouthwashes also have any further influential effects on these properties or not.

It is quite certain that the premature fracture of orthodontic ligature ties, as well as any other dental appliances, would increase the frequency of patient visits to the dentist and consequently affect the patients, both physically and economically. Therefore, this study aims to investigate the mechanical properties, molecular structure, and microscopic morphology of the commercial polymeric orthodontic ligature ties before and after rinsing with commercial mouthwashes. Potential surface interactions between mouthwash and ligature ties were also discussed, based on the obtained results. It is believed that the findings from this study would be useful for the development and/or consideration of mouthwashes for dental patients in future to prevent the premature fractures of polymeric dental appliances.

## 2. Materials and Methods

### 2.1. Sample Preparation

**Figure 1:**
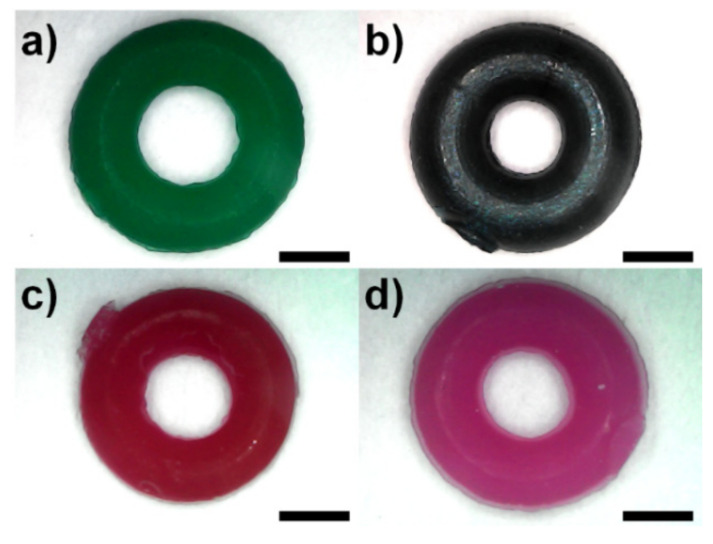
Representative top-view images of the commercial orthodontic elastic ligature ties used in this study: (**a**) Brand A (Dentsply Sirona); (**b**) Brand B (Dent-Mate); (**c**) Brand C (DynaFlex^®^); and (**d**) Brand D (Skyortho Dental Supplies Medical). Scale bar = 1 mm.

Commercial polymeric orthodontic ligature ties from four manufacturers were kindly provided by the Department of Orthodontics, Faculty of Dentistry, Mahidol University. Samples were cut from the casting template and rinsed with commercial mouthwashes at 35–40 °C for 1 h using ultrasonic water bath heater (DT 31 H, BANDELIN, Berlin, Germany). Top-view images of as-received samples were taken by digital USB microscope (Model X4, Shenzhen Haiweixun Electronics Co., Ltd., Shenzhen, China), as shown in Figure 1. The inner and outer diameters of the samples were measured using ImageJ 1.52a software [23], whilst the thickness was measured using digital micrometer (3101-25A, INSIZE, Suzhou New District, China). Commercial mouthwashes used in this study were purchased from three manufacturers, the listed ingredients of which are shown in Table 1.

### 2.2. Universal Tensile Testing

**Figure 2:**
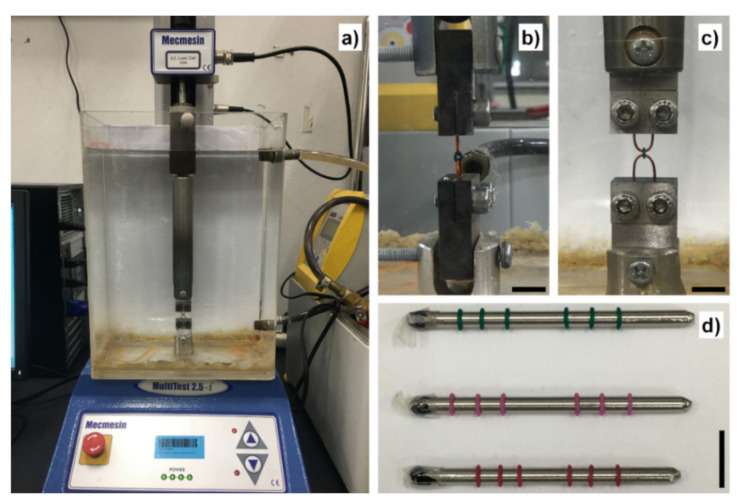
(**a**) The universal tensile testing setup in this study. (**b**) Side-view image of the sample holder. (**c**) Front-view image of the sample holder. (**d**) The samples during uniform stretching experiment. Brand B samples fractured upon being uniformly stretched. Scale bars are approximately 10 mm.

Experiments were performed using universal tensile testing machine with 25 N load cell (MultiTest 2.5-i, Mecmesin, West Sussex, UK) at testing speed of 5 mm/min, as reported previously by Kovatch, et al. [24] and Nakhaei, et al. [21]. Testing temperature was set at 37 °C by submerging the sample holders in the acrylic tank filled with circulated water from the external water bath (Alpha A 6, LAUDA, Lauda-Königshofen, Germany) adapted from Higa, et al. [5], as shown in Figure 2a. Samples were installed to the testing machine by hooking the ligature ties with 20 AWG copper wires clamped to the stainless-steel holders (30 × 20 × 10 mm^3^, height × width × thickness) with two A2-70 stainless-steel M5 screws, as shown in Figure 2b,c. The initial pre-load was set at 0.1 N to ensure that there was minimal clearance prior to the test, and tensile load was applied until the sample fractured.

### 2.3. Fourier-Transform Infrared Spectroscopy (FTIR)

The FTIR transmittance of samples were measured by attenuated total reflectance FTIR spectrometer (FT/IR-4600, JASCO Corporation, Tokyo, Japan). Wavenumber was varied from 500 to 4000 cm^−1^ at around 1 cm^−1^ increment. Data were analyzed using Spectra Manager^TM^ 2.15.01 software (JASCO Corporation, Tokyo, Japan). Measurements were carried out from the as-received samples and the samples rinsed with mouthwashes.

### 2.4. Uniform Stretching Test

Tensile displacement was applied to the as-received samples and the samples rinsed with Mouthwash 2 until reaching 1 mm at 5 mm/min speed and returned to zero-force position at the same speed, by which the delivery force data of the first deformation cycle before uniform stretching were obtained. Subsequently, samples were uniformly stretched by skewering with 3 mm circular stainless steel rods, as shown in Figure 2d, kept at 35–40 °C for 24 h. Tensile displacement was applied to the stretched samples in a similar manner to the first cycle to obtain the delivery force data after uniform stretching.

### 2.5. Scanning Electron Microscopy (SEM)

Representative as-received sample and the sample rinsed with Mouthwash 2 were coated with gold using sputter coater (108auto, Cressington Scientific Instruments Ltd., Watford, UK). Images were taken by scanning electron microscope (JSM-6610 LV, JEOL Ltd., Tokyo, Japan) at 25× and 2500× using the accelerating voltage of 10 kV.

### 2.6. Statistical Analysis

Data were statistically analyzed by using SPSS^®^ Statistics 26 software (IBM^®^, Armonk, NY, USA). Sample size and testing methods are indicated in the figure captions. *p*-values of less than 0.05 were considered as statistically significant.

## 3. Results

### 3.1. Sample Geometry

Table 2 shows the measured geometries of commercial orthodontic ligature ties used in this study. The average inner diameter of Brand A, B, C, and D samples was 1.476, 1.160, 1.292, and 1.334 mm, respectively, whereas the average outer diameter was 3.318, 3.206, 3.061, and 3.081 mm for Brand A, B, C, and D, respectively. On the other hand, the average thickness of Brand A, B, C, and D was 0.778, 0.762, 0.716, and 0.835 mm, respectively.

### 3.2. Universal Tensile Testing

**Figure 3:**
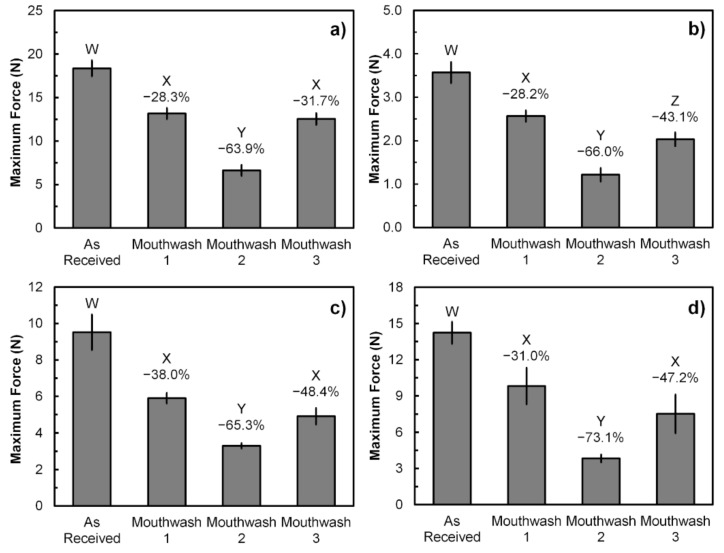
The maximum force obtained from universal tensile testing of the as-received and mouthwash-rinsed samples: (**a**) Brand A; (**b**) Brand B; (**c**) Brand C; and (**d**) Brand D. Error bars represent SD (*n* = 3). Different W, X, Y, and Z letters between the sample groups represent *p* < 0.05 (One-way ANOVA with Tukey’s HSD pairwise comparison) when compared against each other. Reduction percentages are relative to the As-Received samples.

The maximum forces obtained from universal tensile testing of polymeric orthodontic ligature ties before fracture are shown in Figure 3. The average maximum forces bearable by the samples from Brand A, B, C, and D were 18.4, 3.6, 9.5, and 14.2 N, respectively. Moreover, the results also show that rinsing with commercial mouthwashes significantly reduced the maximum force bearable by the samples. Mouthwash 2 has shown the greatest maximum force reduction with the percentage between 63.9 and 73.1, depending on the brand of samples. On the other hand, the maximum force reduction by Mouthwash 1 and 3 are not significantly different from each other with the various percentage between 28.3 and 48.4 across Brand A, C, and D, except for the Brand B samples, of which the maximum force reduction by Mouthwash 3 was greater than Mouthwash 1 at 43.1 and 28.2%, respectively.

**Figure 4:**
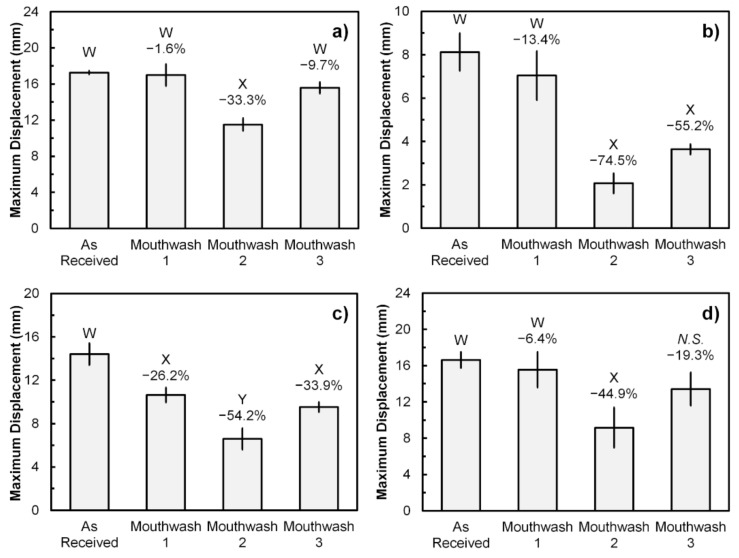
The maximum displacement obtained from universal tensile testing of the as-received and mouthwash-rinsed samples: (**a**) Brand A; (**b**) Brand B; (**c**) Brand C; and (**d**) Brand D. Error bars represent SD (*n* = 3). Different W, X, and Y letters between the sample groups represent *p* < 0.05 (One-way ANOVA with Tukey’s HSD pairwise comparison) when compared against each other. *N.S.* represents *p* > 0.05 when compared with either W or X. Reduction percentages are relative to the As-Received samples.

In the case of the maximum displacement, it was found that Mouthwash 2 has significantly reduced the maximum displacement bearable by all ligature ties, as shown in Figure 4. The average maximum displacement of samples from Brand A, B, C, and D were 17.3, 8.1, 14.4, and 16.6 mm, respectively. The displacement reduction percentage influenced by Mouthwash 2 was varied between 33.3 and 74.5%. On the other hand, Mouthwash 1 only reduced the maximum displacement of Brand C samples significantly by 28.2%, whereas Mouthwash 3 has significant effects on both Brand B and C samples by 55.2 and 33.9%, respectively. However, it was also noted that the influence of Mouthwash 3 on Brand D samples was not significantly different from both as-received and Mouthwash 2 samples in terms of their maximum displacement.

### 3.3. Fourier-Transform Infrared Spectroscopy (FTIR)

**Figure 5:**
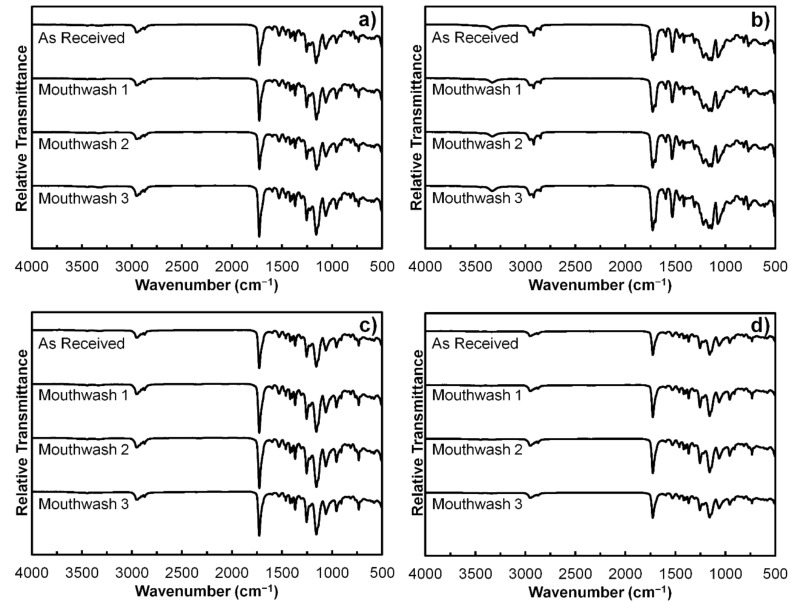
The FTIR spectra of the as-received and mouthwash-rinsed samples: (**a**) Brand A; (**b**) Brand B; (**c**) Brand C; and (**d**) Brand D.

Figure 5 shows the FTIR spectra of polymeric orthodontic ligature ties from all four brands, including both as-received and rinsed samples. It was found that all samples exhibited peaks at 2955, 1725, 1595, 1530, 1460, 1415, 1310, 1220, 1160, and 955 cm^−1^, which corresponded with the organic components of their structure, including O–H, C=O, N–H, C–H, and C–O [25,26,27]. Moreover, peaks at 3400, 1700, 1075, and 730 cm^−1^ were present in Brand B samples, but not the other three brands. This could be due to the presence of different classes of N–H, C=O, C–O, and C–H bonds in their structure [25,26,27]. On the other hand, although the peak at 1530 cm^−1^ was present in all samples, the depth of the peak was relatively greater in Brand B samples than those of the other three brands. It was also noticeable that the shape of the FTIR spectra of Brand A, C, and D samples were consistent among each other, whereas the spectra of Brand B samples were distinct. In addition, the difference in the FTIR spectra between the as-received and rinsed samples were also not observable from the samples of every brand.

### 3.4. Uniform Stretching Test

**Figure 6:**
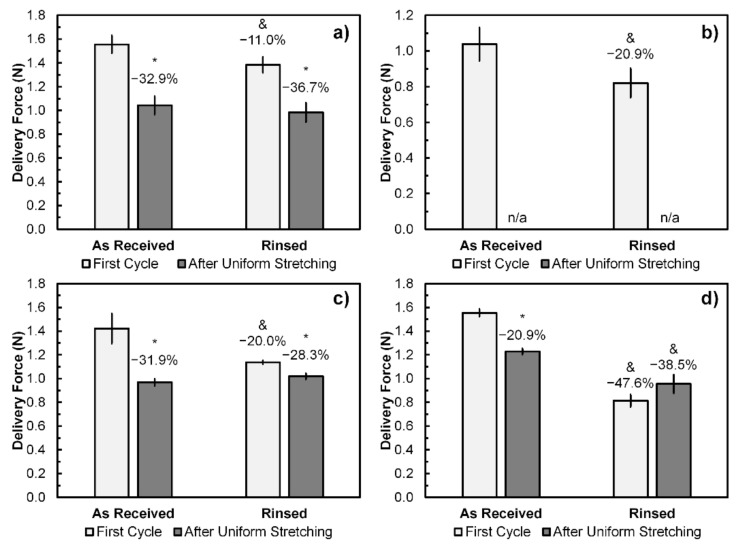
Delivery force at 1 mm displacement obtained from universal tensile testing of the as-received and Mouthwash-2-rinsed samples at the first deformation cycle and after uniform stretching: (**a**) Brand A; (**b**) Brand B; (**c**) Brand C; and (**d**) Brand D. Error bars represent SD (*n* = 3). * represents *p* < 0.05 (Paired two-tailed Student’s *t*-test) when compared with its first testing cycle. & represents *p* < 0.05 (Unpaired two-tailed Student’s *t*-test) when compared with the As-Received samples at the same testing cycle. n/a indicates that the samples fractured prior to the delivery force measurement after uniform stretching. Reduction percentages are relative to the first cycle of the As-Received samples.

Since Mouthwash 2 has shown the most significant effects on both maximum forces and displacement, it was selected for the following experiments to investigate further on the changes in delivery forces and morphologies. Delivery forces were obtained from the as-received and rinsed samples by applying tensile displacement of 1 mm. It is noted that the samples from Brand B have fractured upon being uniformly stretched and thus their delivery forces after uniform stretching could not be obtained. The results from this experiment are shown in Figure 6, from which it can be seen that the average delivery force at the first deformation cycle of Brand A, B, C, and D samples were 1.55, 1.04, 1.42, and 1.55 N, respectively. Furthermore, rinsing with Mouthwash 2 has significantly reduced the delivery force at the first deformation cycle of orthodontic ligature ties. The reduction percentage varied between 11.0 and 47.6% across all four brands, of which Brand D exhibited the greatest delivery force reduction. 

Moreover, apart from the premature fractures of Brand B samples, it was also found that the delivery forces after uniform stretching were significantly lower than the first cycle in both as-received and rinsed samples, except for the rinsed Brand D samples that the difference between delivery force at the first cycle and after uniform stretching was not statistically significant. The average delivery forces after uniform stretching of the as-received Brand A, C, and D samples were 1.04, 0.97, and 1.23 N, which correspond to the reduction percentages of 32.9, 31.9, and 20.9%, respectively. However, differences in delivery forces after uniform stretching between the as-received and rinsed ligature ties were not significant in Brand A and C samples, whereas those of rinsed Brand D samples were less than the as-received samples.

### 3.5. Scanning Electron Microscopy (SEM)

**Figure 7:**
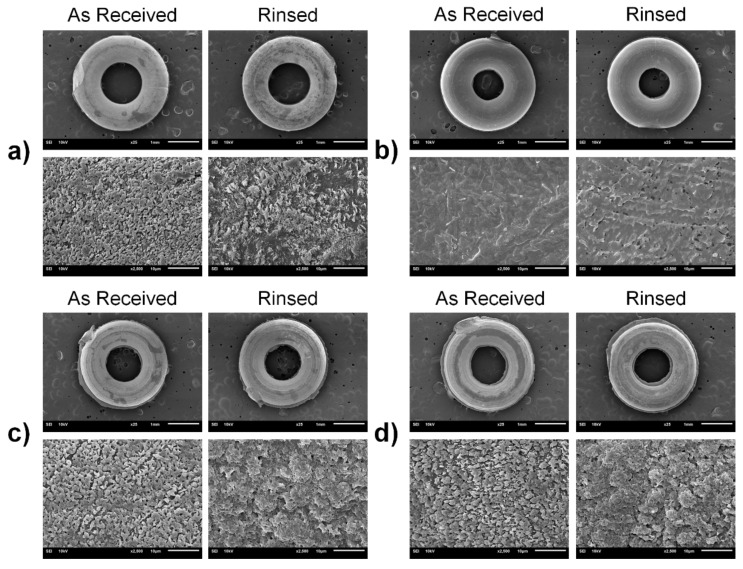
Representative top-view images of the samples taken by scanning electron microscope (SEM) of the as-received sample and the sample rinsed with Mouthwash 2: (**a**) Brand A; (**b**) Brand B; (**c**) Brand C; and (**d**) Brand D. Top-row images of each brand were taken at 25× (Scale bar = 1 mm). Bottom-row images of each brand were taken at 2500× (Scale bar = 10 μm).

Surface of the as-received samples and those rinsed with Mouthwash 2 are shown in Figure 7, both at 25× and 2500× magnifications. It is clearly distinguishable that rinsing with Mouthwash 2 altered the surface morphology of the samples. For instance, the rinsed surface of Brand A, C, and D samples appeared to be more aggregated and coarser than those of the as-received samples, whereas porous defects were observed on the rinsed surface of Brand B samples. Likewise, it was also noticeable that the surface of Brand B ligature tie was relatively smoother than the others.

## 4. Discussion

It is seen from the results that rinsing the polymeric orthodontic ligature ties with commercial mouthwashes has significantly altered their mechanical properties, which could further affect the efficacy of orthodontic treatments to some extent. Furthermore, the findings from this study also provide information to discuss the primary interactions behind the observable changes in mechanical properties, the details of which are further discussed in the following sections.

### 4.1. Changes in Molecular Structure and Microscopic Morphology of Polymeric Orthodontic Ligature Ties

FTIR is one of the techniques that characterize the organic and inorganic constituents of the samples, thereby providing the information on their molecular structure [25,26]. Class of the functional groups can be determined based on the presence of peaks at specific wavenumbers [25,26]. From the results, shapes of FTIR spectra indicate that Brand A, C, and D ligature ties were made of similar materials and were different from those of Brand B. Based on the fact that the material components and additives of each brand are proprietary, it is impractical to precisely identify the material type of four ligature tie brands using the FTIR spectra alone, although they are likely to be of polyurethane family [1,28,29]. Nonetheless, it is understood from the FTIR spectra that rinsing with three commercial mouthwashes did not alter the molecular structure of polymeric orthodontic ligature ties as there was no significant change in the presence of peaks from the as-received samples. In the case of microscopic morphology, noticeable changes on the sample surface after mouthwash rinsing were observable from the SEM images, either the topographical changes or the presence of porous defects. However, it is still unclear whether the associated reactions were oxidative, reductive, hydrolytic, or dissolution, and this requires further experiment to investigate the chemical interactions between mouthwash and the ligature ties in detail [30]. These findings suggest that the alteration of molecular structure may not be the cause of the changes in mechanical properties of polymeric orthodontic ligature ties after rinsing with mouthwashes, but the microscopic morphology could be the case.

### 4.2. Reduction in the Maximum Force, Maximum Displacement, and Delivery Force of Polymeric Orthodontic Ligature Ties

The results have shown that each brand of polymeric orthodontic ligature ties exhibited different maximum bearable forces and displacement, even without rinsing with mouthwashes. In general, there are two major factors that contributed to these differences: the sample geometry; and the materials. Sample geometry influences the maximum force and displacement in a way that it dictates the mechanical stress and strain induced by deformation [31]. In the case of polymeric orthodontic ligature ties, samples with greater average diameter would require higher force to break [31]. However, even then, the maximum force of Brand B samples was the lowest, despite not being the smallest in the average diameter. This was due to the fact that Brand B samples had the shortest internal circumferences, which increased the mechanical strain per unit of displacement of samples [31]. Hence, the localized concentration of mechanical stress and strain around the internal circumference area was likely to be the cause of fracture for Brand B samples, and this could also be the reason why Brand B samples exhibited the shortest maximum displacement and fractured upon being uniformly stretched. In addition to the sample geometry, the second factor that affects mechanical properties is the material. It is seen from the FTIR spectra that Brand B samples were made of different material from the other three brands, and this may also contribute to the lower maximum force and displacement bearable by Brand B samples.

Furthermore, it is also found from the results that rinsing the polymeric orthodontic ligature ties with mouthwashes significantly reduced the maximum force and displacement of the samples. In other words, it made the ligature ties weaker and more fragile, and thus it could negatively affect the tooth movement and efficacy of orthodontic treatments [4,5]. Despite the difference in maximum forces and displacement, the average delivery forces at 1 mm displacement from the ligature ties were consistent among Brand A, C, and D, ranging from 1.42 to 1.55 N. On the contrary, Brand B samples deliver lower average force of 1.04 N at 1 mm displacement. These findings imply that the sample geometry and/or materials have also influenced the delivery forces of polymeric orthodontic ligature ties. However, it should also be pointed out that there are a wide range of commercially available polymeric orthodontic ligature ties with various reported delivery forces, ranging between 0.7 and 5.3 N [21,22].

The delivery forces from polymeric orthodontic ligature ties were found to decrease following uniform stretching, which mimicked the early stage of their usage [21]. This was due to the viscoelastic properties of elastomer that the material would relax when experiencing constant stress beyond its stress relaxation time, whereby the deformation became permanent and thus reduced the delivery force [1,2,3,5,24,32,33]. It is evident that the relaxation time of polymeric orthodontic ligature ties used in this study was shorter than 24 h, at which the delivery force had already dropped by between 11.0 and 47.6% following uniform stretching. This range of reduction percentage overlapped with previous studies in which the delivery force reduction ranging between 30 and 50% were reported after applying constant uniform stretching or tension [21,22]. However, mouthwash rinsing seemed to be less influential on the level of delivery force after uniform stretching. In addition, the delivery force reduction due to viscoelasticity was also observed in other polymeric orthodontic appliances, including elastic chains and bands [34,35,36,37].

### 4.3. Potential Surface Interactions between Mouthwash and Polymeric Orthodontic Ligature Ties and Future Studies

According to the results, it is plausible that mouthwash has affected the mechanical properties of ligature ties through microscopic surface interactions, as seen from the SEM images. More aggregated and coarser surfaces were observed from the samples rinsed with Mouthwash 2, and also the porous defects in the case of Brand B samples. These changes in surface properties could negatively affect the mechanical properties of the samples [38,39]. pH of mouthwashes could be one of the influential factors as it has been reported that the maximum forces, displacement, and delivery forces of some commercial elastic bands were significantly reduced upon the exposure to NaOH, which is a strong alkaline solution [37]. Despite that, this effect was not significant when observed at acidic and neutral pH [40]. Furthermore, a recent study has demonstrated that the exposure to commercial mouthwash reduced the tensile strength of nitrile O-rings used for overdenture attachment [20]. Changes in surface morphology following the exposure to a certain type of cleaning solution were also reported [20]. However, the influence of bleaching agents at the concentration used in commercial mouthwashes appeared to be not as significant, based on the findings from another study [41]. The presence of alcohol can also play a role, as the alcohol-containing mouthwash exhibited greater force reduction in orthodontic elastomeric chains than the alcohol-free type [42,43]. Consistently, alcohol was only contained in Mouthwash 2 in this study, and thus this could be the reason why it exhibited the greatest effects on mechanical properties of ligature ties. In addition, the increased surface roughness of ligature ties by mouthwash rinsing also potentially affects their tribological properties in terms of friction and wear during their actual operations [32,44].

This study has highlighted that the use of commercial mouthwashes has significantly affected the mechanical properties of orthodontic appliances, despite being able to improve the hygiene of oral cavity. Hence, it would need further consideration regarding which type of commercial mouthwashes is suitable for orthodontic patients who use polymeric appliances. Moreover, it would be worth investigating further in terms of the ligature tie discoloration influenced by commercial mouthwashes as these aesthetic issues are also concerned when installing the orthodontic appliances [21,45,46,47]. Likewise, the effects of long-term or repeated exposure to mouthwash on the properties of ligature ties were not covered in this study, though it was reported that the delivery force can reduce continuously for up to eight weeks during their operation [21,22]. In the case of ligature tie geometry, finite element technique may also be used for size optimization to achieve the optimal mechanical performance whilst minimizing the amount of materials used [4,10]. Finally, it is worth mentioning that the inherited properties of commercial orthodontic ligature ties also include cytotoxic effects [48]. Viability of L-929 cells upon the exposure to ligature ties were reported to be between 51 and 93% across three different commercial brands, one of which noticeably exhibited significant cytotoxic effects [48]. Therefore, it may be of interest to the future study to investigate whether or not mouthwash rinsing could influence the cytotoxicity of orthodontic ligature ties.

## 5. Conclusions

It can be concluded from this study that the use of commercial mouthwashes has affected the mechanical properties of polymeric orthodontic ligature ties, which could consequently affect the efficacy of orthodontic treatments. Maximum bearable forces and displacement were significantly reduced by mouthwash rinsing, as well as the initial delivery forces. Changes in microscopic morphology were plausibly one of the major causes behind the observable changes in the ligature tie properties influenced by mouthwash rinsing. Therefore, the type of commercial mouthwashes may need to be taken into consideration, especially for the orthodontic patients who are using polymeric appliances.

## Figures and Tables

**Table 1:** Details of the commercial mouthwashes used in this study.

Labels	Ingredients Listed on the Label			
Mouthwash 1 (Colgate^®^ Plax^®^ Peppermint, Bangkok, Thailand)	Water	Sodium fluoride	Poloxamer 407	Menthol
	Flavor	Glycerin	Propylene glycol	Sorbitol
	Sodium saccharin	Cetylpyridinium chloride	Potassium sorbate	CI 42051
Mouthwash 2 (Listerine^®^ Cool Mint, Bangkok, Thailand)	Water	Benzoic acid	Poloxamer 407	Menthol
	Flavor	Methyl salicylate	Eucalyptol	Sorbitol
	Sodium saccharin	CI 42053	Thymol	Alcohol
	Sodium benzoate			
Mouthwash 3 (Fluocaril^®^, Bi-Fluoré Ortho 123, Bangkok, Thailand)	Water	Sodium fluoride	PPG-26 Buteth-26	Xylitol
	Flavor	Glycerin	PEG-40 Hydrogenated Castor Oil	Panthenol
	Sodium saccharin	Cetylpyridinium chloride	Sodium Monofluorophosphate	CI 14720
	Sodium benzoate	*Aloe barbadensis* leaf extract	*Centella asiatica* extract	CI 16255
	Citric acid			

**Table 2:** Measured geometries of the commercial orthodontic ligature ties used in this study.

Labels	Inner Diameter (mm) Mean ± SD (*n* = 5)	Outer Diameter (mm) Mean ± SD (*n* = 5)	Thickness (mm) Mean ± SD (*n* = 5)
Brand A (Dentsply Sirona, Charlotte, North Carolina, USA)	1.476 ± 0.083	3.318 ± 0.151	0.778 ± 0.008
Brand B (Dent-Mate Co., Ltd., Bangkok, Thailand)	1.160 ± 0.045	3.206 ± 0.120	0.762 ± 0.004
Brand C (DynaFlex^®^, St. Ann, Missouri, USA)	1.292 ± 0.040	3.061 ± 0.084	0.716 ± 0.009
Brand D (Skyortho Dental Supplies Medical Co., Ltd., Yancheng, China)	1.334 ± 0.118	3.081 ± 0.199	0.835 ± 0.007

## Data Availability

The datasets generated during this study are available from the corresponding author on reasonable request.
